# Transcranial ultrasound stimulation applied in ischemic stroke rehabilitation: A review

**DOI:** 10.3389/fnins.2022.964060

**Published:** 2022-07-22

**Authors:** Jiecheng Guo, Wai Leung Ambrose Lo, Huijing Hu, Li Yan, Le Li

**Affiliations:** ^1^Institute of Medical Research, Northwestern Polytechnical University, Xi’an, China; ^2^Department of Rehabilitation Medicine, The First Affiliated Hospital, Sun Yat-sen University, Guangzhou, China

**Keywords:** transcranial ultrasound, stroke, thrombosis, intervention, drug delivery

## Abstract

Ischemic stroke is a serious medical condition that is caused by cerebral vascular occlusion and leads to neurological dysfunction. After stroke, patients suffer from long-term sensory, motor and cognitive impairment. Non-invasive neuromodulation technology has been widely studied in the field of stroke rehabilitation. Transcranial ultrasound stimulation (TUS), as a safe and non-invasive technique with deep penetration ability and a tiny focus, is an emerging technology. It can produce mechanical and thermal effects by delivering sound waves to brain tissue that can induce the production of neurotrophic factors (NFs) in the brain, and reduce cell apoptosis and the inflammatory response. TUS, which involves application of an acoustic wave, can also dissolve blood clots and be used to deliver therapeutic drugs to the ischemic region. TUS has great potential in the treatment of ischemic stroke. Future advancements in imaging and parameter optimization will improve the safety and efficacy of this technology in the treatment of ischemic stroke.

## Introduction

Stroke is one of the major causes of chronic disability worldwide (Zhu et al., 2022). Stroke can be classified as ischemic or hemorrhagic stroke, with ischemic stroke accounting for approximately 75% of all stroke cases (Donkor, 2018). This cerebrovascular condition can cause a considerable number of functional limitations and can lead to death in severe cases (Sousa et al., 2009). In the clinic, stroke treatment often involves thrombolysis and surgical recanalization (Paul and Candelario-Jalil, 2021). Thrombolytic therapy includes drug thrombolysis and interventional thrombectomy. At present, intravenous thrombolysis has certain limitations. For example, the classic treatment, i.e., intravenous injection of tissue plasminogen activator (tPA), has a short treatment window, and the risk of bleeding complications is high. tPA is also not suitable for patients with comorbidities, such as bleeding, hypertension and those who are on anticoagulation therapy. The clinical application of interventional is limited by technical challenges, equipment requirements and high cost (Powers et al., 2019). Therefore, research on safe and effective new treatment approaches for promoting nerve recovery after stroke is of great significance.

Non-invasive neuromodulation techniques, including transcranial magnetic stimulation (TMS), transcranial direct current stimulation (tDCS) and transcranial alternating current stimulation (tACS), have been applied in clinical settings to promote neural plasticity and improve function in stroke patients (Cantone et al., 2021). TMS and tDCS have different effects on neurons (Woods et al., 2016). TMS modulates neuronal excitability by generating a magnetic field that induces neuronal depolarization (Antczak et al., 2021), and it can also cause vascular cognitive impairment, which may lead to dementia (Cantone et al., 2020; Di Lazzaro et al., 2021). tDCS and tACS can modulate cortical excitability by hyperpolarization or depolarization neuronal resting membrane potentials (Pol et al., 2021). However, Airan reported that the absorption and scattering of magnetic and electrical signals within the brain limits the spatial resolution and penetration depth of these techniques, which further hinders their application for the treatment of stroke (Airan, 2017). Studies have found that transcranial focused ultrasound stimulation (TFUS), a non-invasive, high resolution and safe technology, can modulate neural activity and exert neuroprotective effects (Li et al., 2016; Baek et al., 2018). Ultrasound stimulation has been used to treat cancer, neurodegenerative diseases, diabetes and thrombosis (Miller and O’Callaghan, 2017; Tharkar et al., 2019; Zhang et al., 2019; Ma et al., 2021). Low-intensity focused ultrasound stimulation (LIFUS) has been shown to be efficacious in the treatment of a variety of neurological and psychiatric disorders (Li et al., 2017; Wang et al., 2022b) such as Parkinson’s disease (PD), Alzheimer’s disease (AD) epilepsy and stroke, and animal experiments have shown that LIFUS does not cause any tissue damage when used to treat stroke, proving that transcranial ultrasound stimulation (TUS) in the treatment of ischemic stroke has good research prospects and has the potential to become a non-invasive treatment method (Min et al., 2011; Liu et al., 2019; Shin et al., 2019; Ma et al., 2021).

This narrative review provides an overview of the current literature on the application of TUS in animal models and human subjects, discusses the potential effects of TUS in the treatment of ischemic stroke and the various treatment methods, and provide further theoretical and technical support for the clinical application of TUS.

## Transcranial focused ultrasound stimulation technology and principle

The applications of focused ultrasound have been increasing, and diagnostic ultrasound has been established as a critical clinical imaging modality (Trockel et al., 1984; Fu et al., 2015; Nainwal, 2017; Darrow, 2019; di Biase et al., 2021; Liu et al., 2021). TFUS is a non-invasive technology that can be used to monitor cerebral circulation (Thomassen et al., 2021). Spatially limited energy can be delivered to brain tissue at a wide range of intensities, allowing high temporal resolution and spatial visualization of the intracranial and extracranial arteries (Sharma et al., 2011). In addition, TFUS can be used to enhance or inhibit nervous system activity by adjusting the frequency (Kubanek, 2018b), intensity and stimulation time (Kubanek, 2018b; Tyler et al., 2018).

Transcranial focused ultrasound stimulation waves are transmitted to human tissue in a continuous or pulsed form through an ultrasonic probe (Zhang et al., 2022). They can modulate neural excitability (Kamimura et al., 2020) by producing mechanical effects (Blackmore et al., 2019) through alterations in ion channels (Yoo et al., 2011), membrane capacitance (Plaksin et al., 2014), the generation of sonopores (Tata and Dunn, 1992) and interfacial elastic wave coupling, and by producing thermal effects through the rise in temperature caused by sound waves (Cesare et al., 1999; Kamimura et al., 2020), thus, ultrasonic stimulation is a potential non-invasive treatment for neurological diseases. The therapeutic effect of TUS is mainly influenced by the carrier frequency, peak intensity, duration, pulse repetition frequency and duty cycle (Kubanek, 2018a). Ultrasound stimulation at different parameters induces different therapeutic effects. TFUS can utilize ultrasound phased array technology to electronically drive an ultrasound transducer array to direct focused ultrasound beams to different neural targets, enabling large-scale ultrasound neuromodulation within a given tissue volume (Monteith et al., 2013; Ilham et al., 2021), it can also transmit acoustic energy to a target area in the brain through single-element transducers, acting on a focal point (Park et al., 2022). LIFU (frequency:<1 MHz; intensity: 0.5-100 W/cm^2^) (Tyler et al., 2018) stimulates nerve tissue mainly through the pressure generated by ultrasonic radiation, improving the blood supply around the brain lesion by means of neural regulation without causing tissue damage (Bystritsky et al., 2011; Fomenko et al., 2018; Wang et al., 2022a). Therefore, LIFU is a good option for non-invasive treatment of ischemic stroke.

## The effects of transcranial ultrasound stimulation in stroke rehabilitation

### Promotion of neurological recovery

It is well known that some important neural factors, such as vascular endothelial growth factor (VEGF) and brain-derived neurotrophic factor (BDNF), have a protective effect against ischemic brain injury and are potential therapeutic targets. Angiogenesis is closely related to neurogenesis in the adult mammalian brain (Palmer et al., 2000). VEGF can regulate axonal growth, neuronal survival, and neovascularization (Greenberg and Jin, 2005). BDNF is a neurotrophin that is widely expressed in the central nervous system and plays a key role in memory, the differentiation and survival of neurons and synaptic plasticity (Bartkowska et al., 2007; Balkaya and Cho, 2019). Low-intensity pulsed ultrasound stimulation (LIPUS) has been proven to have a neuroprotective effect on brain injury (Bretsztajn and Gedroyc, 2018). Short-term application of transcranial pulse ultrasound stimulation can increase in the density of BDNF-positive lacrimal spots in the hippocampus, suggesting that ultrasound can stimulate hippocampal neuronal activity and promote endogenous brain plasticity (Tufail et al., 2010). LIPUS can also increase the expression of BDNF and VEGF in astrocytes and inhibit cell apoptosis (Yang et al., 2015; Su et al., 2017). LIPUS was applied to the brains of mice before middle cerebral artery occlusion (MCAO)-induced cerebral ischemic injury and was found to significantly reduce neuron apoptosis of neurons in brain tissue, prevent a decline in cell viability, ameliorate neuronal injury, and alleviate ischemic stroke (Chen et al., 2018). Application of LIPUS for 9 consecutive days before secondary MCAO was shown to reduce mortality, attenuate pathological changes, and significantly reduce neuronal apoptosis in mice with recurrent stroke (Wu et al., 2019). Postischemia angiogenesis regulates axon growth and neurogenesis, including the proliferation, migration, maturation of nerve stem/progenitor which contribute to functional recovery (Taguchi et al., 2015). Ultrasound has the ability to vascularize endothelial cells (ECs) and promote angiogenesis (Imashiro et al., 2021). Application of LIPUS for 20 min a day for 4 weeks was shown to upregulate VEGF expression and enhance endothelial nitric oxide synthase (eNOS) activity *in vitro* (Hanawa et al., 2014). LIPUS was a found to significantly increase the number of peripheral CD31-positive blood vessels and ischemic striatum doublecortin (DCX)-positive neurons, and the gene expression levels of eNOS, VEGF, and fibroblast growth factor. Activated ECs secrete VEGF to increase neurogenesis. LIPUS is likely to promote the proliferation and migration of neural stem cells by establishing suitable blood vessels as scaffolds, and promote the repair of brain injury after stroke by inducing expression of a series of neurotrophic factors (NFs) (Ichijo et al., 2021).

**FIGURE 1:**
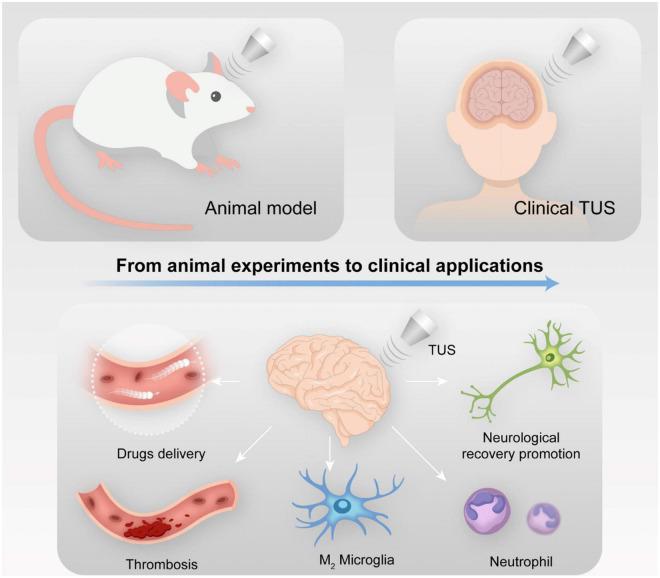
The mechanism of transcranial ultrasound stimulation (TUS) in stroke rehabilitation.

Transcranial ultrasound stimulation can increase the expression of BDNF and VEGF, which play a protective role during brain injury rehabilitation, in the brain. Thus, TUS may be applied a novel approach for clinical stroke treatment (Figure 1).

### Amelioration of inflammation

Oxygen and energy consumption after ischemic stroke trigger a cascade of damage, including an inflammatory response that leads to severe brain damage. After ischemic stroke, microglia are activated and produce both harmful and neuroprotective mediators. The balance between these types of mediators determines the outcome of neuronal damage (Zhao et al., 2017). Any disruption or loss of cerebral homeostasis with real or potential effects on the central nervous system causes rapid and intense changes in microglial shape, gene expression, and function. These changes are known as microglial activation (Kettenmann et al., 2011). When brain injury occurs, microglia become activated and are polarized toward the M1 or M2 phenotype. Inflammatory M1 microglia secrete inflammatory factors, which may exacerbate autologous brain injury in early ischemic stroke, and M2 microglia secrete the anti-inflammatory cytokine IL-10, which in turn can induce the polarization of microglia toward the M2 phenotype. IL-10 is an important immunomodulator in the central nervous system and a key factor in poststroke recovery. It can not only suppress the inflammatory response but also alter neurogenesis and promote synaptic remodeling (Wang et al., 2021). LIPUS can significantly reduce the levels of TNF-α, IL-1β, and IL-6 in microglia, and inhibit the expression of proinflammatory mediators in microglia and microglial apoptosis to control the inflammatory response (Chang et al., 2020). In a previous study, TUS was applied to stimulate the ischemic hemisphere in mice 1 week after cerebral ischemia. Microglia in the ischemic brain area were polarized toward the M2 phenotype, the expression levels of IL-10R and IL-10 in the brain were increased, the cerebral infarct volume was significantly reduced, and neurological severity scores and behavioral scores were improved (Wang et al., 2021). Ultrasound can induce the polarization of microglia toward the M2 phenotype through the IL-4 signaling pathway, protect against brain injury in mice and promote functional recovery by alleviating nerve function defect, inhibiting nerve cell apoptosis and reducing the destruction of the blood-brain barrier (BBB). It can also promote tissue reconstruction and nerve regeneration. Engineered platelet-fused microglia can achieve functional neuronal regeneration after ischemic stroke (Li et al., 2021). After stroke, the number of neutrophils increases rapidly, which leads to the disruption of the BBB, cerebral edema, and brain damage. In response to the mutual interaction between neutrophils and the cerebral endothelium, a large amount of neutrophil-derived reactive oxygen species, proteases and cytokines are released in the brain (Jickling et al., 2015). Reactive microglia surrounding the infarct can engulf neutrophils, preventing neutrophils from accumulating in this area (Otxoa-de-Amezaga et al., 2019). Applying low-intensity pulsed transcranial ultrasound stimulation (PTUS) with a frequency of 0.5 MHz to the ischemic cortex after distal occlusion of the middle cerebral artery can reduce the number of neutrophils in the damaged area and the inflammatory response in the brain (Guo et al., 2015).

TUS and LIPUS can modulate neural activity in different brain regions by affecting the polarization of microglia in the brain. TUS and LIPUS can induce the polarization of microglia toward the protective phenotype and reduce the detrimental effect of neutrophil injury; thus, they are effective in reducing inflammation after stroke.

### Thrombolysis

Transcranial focused ultrasound stimulation can improve or restore blood flow in blood vessels blocked by a clot (Birnbaum et al., 1998). However, an important factor to be considered in ultrasound thrombolysis is the size and concentration of the clot fragments, as they may block the micro vessels in the distal vascular bed. It is generally believed that the efficacy of ultrasound thrombolysis is based on its cavitation effect, which facilitates the dissolution of thrombi (Table 1). Under ultrasonic irradiation, tiny bubbles in the blood vibrate and grow under the action of the ultrasonic field and continuously gather the energy of the sound field; when the energy reaches a certain threshold, the cavitated bubbles collapse, promoting the dissolution of the thrombus (Kubanek, 2018a). However, TFUS is often used as an auxiliary thrombolytic method to enhance the efficacy of intravenous thrombolytic drugs (Wright et al., 2012). It has been reported that ultrasound at a frequency of 490 kHz and an intensity of 0.13 W/cm^2^ can promote tPA thrombolytic effect and improve blood perfusion (Ishibashi et al., 2002). Ultrasound stimulation at a frequency of 490kHz and an intensity of 0.8 W/cm^2^ can enhance the thrombolytic effect of tPA, significantly reducing the cerebral infarct volume and improving neurological function without causing hemorrhagic complications (Saguchi et al., 2008). A clinical study also confirmed that continuous TUS can enhance tPA-induced arterial recanalization (Alexandrov et al., 2004). In addition to thrombolytic drugs, microbubble-assisted thrombolysis can greatly enhance the effect of thrombolytic therapy, and there is no difference in effectiveness between microbubble-assisted thrombolysis and thrombolytic therapy involving different microbubbles (Schleicher et al., 2016). Ultrasound combined with microbubbles can effectively remove thrombi when combined with a very low dose of tPA or in the absence of tPA, and significantly reduce the infarct volume without causing obvious side effects (Brown et al., 2011; Culp et al., 2011). High mechanical index (MI) PTUS combined with microbubbles can dissolve thrombi and improve ipsilateral and contralateral cerebral blood flow after acute cerebral embolism (Gao et al., 2014). A combination of microbubbles with transcranial TUS at a frequency of 1 MHz and intensity of 2.0 W/cm^2^ can rapidly resolve acute intracranial thrombotic occlusions (Culp et al., 2004). Thrombolytic efficacy can be further enhanced through the use of targeted microbubbles (TMBs). This may be because adhesion between microbubbles and the thrombus is enhanced and the concentration of bubbles around the thrombosis increased, leading to promotion of the cavitation effect and thus enhancement of the thrombolytic effect. The combination of thrombolytic therapy and recombinant tissue plasminogen activator (r-tPA) is safer than the application of r-tPA alone and decreases the risk of intracranial hemorrhage (Ren et al., 2012). Since the combination of ultrasound and microbubbles has an enhanced thrombolytic effect and does not increase the incidence of cerebral hemorrhage, r-tPA can be administered during thrombolytic therapy (Lu et al., 2016). It has also been reported that the addition of microbubbles enhances the effects of transcranial ultrasound-assisted urokinase thrombolysis, significantly reducing the infarct size without increasing the risk of cerebral hemorrhage (Liu et al., 2012).

**TABLE 1:** Summary of transcranial focused ultrasound in neuromodulation and reduction of inflammatory responses.

References	Affect	Stimulation time	Stimulation area	Stimulus object	Intensity	Frequency	Ultrasound type
Yang et al., 2015	Elevate BDNF, VEGF	15 min, 5 min interval	None reported	Normal SD rat	I_SPTA_ = 528 W/cm^2^	1 MHZ	LIPUS
Su et al., 2017	Elevate BDNF, VEGF	5 min a day, 3 days	Cortex	Cortically damaged mice	I_SPTA_ = 528 W/cm^2^	1 MHZ	
Chen et al., 2018	Elevate BDNF, VEGF, reduce apoptosis	15 min, 5 min interval, 5 days	None reported	MCAO mice	I_SPTA_ = 528 W/cm^2^	1 MHZ	
Wu et al., 2019	Elevate BDNF, reduce apoptosis	15 min, 9 days	Cerebral cortex	Secondary MCAO mice	I_SPTA_ = 528 W/cm^2^	1 MHZ	
Ichijo et al., 2021	Elevate VEGF, eNOs, CD13, DCX	3 times a day, 20 min each time, 3 days	Whole brain	MCAO mice	I_SPTA_ = 193 mW/cm^2^	0.5 MHZ	
Chang et al., 2020	Elevate BDNF, VEGF, reduce TNF-α, IL-1β, IL-6	3 times, 5 min each time	None reported	Vitro cultured glial cell line	I_SPTA_ = 30 mW/cm^2^	1 MHZ	
Wang et al., 2021	Activate microglia, elevate IL-10, IL-10R	10 min a day, 7 days	Ischemic hemisphere	Ephemeral MCAO mice	I_SPPA_ = 120 mW/cm^2^	0.5 MHZ	TFUS
Guo et al., 2015	Reduce neutrophils	60 min	Ischemic core	MCAO rat	I_SATA_ = 86 mW/cm^2^	0.5 MHZ	PTUS

I_SPTA_: (spatial-peak temporal-average intensity). I_SPPA_: (spatial peak pulse average acoustic intensity). I_SATA_: (spatial-average temporal-average intensity). LIPUS (Frequency: 1-3 MHz, Intensity: 0.02–1 W/cm^2^).

The cavitation effect is the main mechanism underlying the effect of ultrasonic thrombolysis. The effectiveness and safety of ultrasonic thrombolysis have also been confirmed by a large number of experimental studies. However, to date, there is no consensus on the optimal parameters of ultrasonic thrombolysis, and further exploration is needed.

### Drug delivery

Ultrasound combined with microbubble-mediated drug delivery is a non-invasive, targeted drug delivery approach that can be guided by imaging technology through the interaction of microbubbles undergoing acoustic cavitation and cells. Delivery of therapeutic substances to the target tissue or organ through an ultrasonic microbubble contrast agent mainly relies on ultrasonic TMB destruction technology. The therapeutic agent is released at a specific point under the action of ultrasonic irradiation for diagnosis or treatment. Oscillation and implosion of the microbubbles result in an increase in temperature (Wood and Sehgal, 2015), shock waves, the shear stress, mechanical stress, and the generation of free radicals (Barnett et al., 1994; Rosenthal et al., 2004; Ashokkumar, 2011; Sanwal et al., 2021). These phenomena are the main mechanisms of ultrasound-enhanced targeted drug delivery (Shin Low et al., 2021). The BBB is a key factor affecting the delivery of drugs to the central nervous system. Ultrasound can induce opening of the BBB and promote drug delivery. Ohta et al. (2020) investigated the effect of size on the ability of nanoparticles to be delivered to the brain *via* FUS-induced BBB opening, using gold nanoparticles (AuNPs) with diameters of 3, 15, and 120 nm. They found that medium-sized (15 nm) AuNPs showed the highest delivery efficiency (0.22% ID). The steady and inertial cavitation doses were quantified by labeling lipid microvesicles with the fluorophore 5-dodecylamino fluorescein and applying FUS to the rat head to aid the transport of lipid microvesicles across the BBB; in this way, the cavitation dose threshold was determined for the first time (Sierra et al., 2017). FUS-mediated delivery of NFs, including BDNF (Baseri et al., 2012), neurturin and GDNF (Wang et al., 2012), has been shown to result in high enough levels of these factors to induce neuroprotection and survival. In the treatment of AD, focused ultrasound combined with microbubbles formed by embedding quercetin-modified sulfur nanoparticles (Qc@SNPs) can induce opening of the BBB and allow the delivery of Qc@SNPs. This can effectively protect nerve cells by reducing neuronal apoptosis, the inflammatory response, calcium homeostasis imbalance and oxidative stress (Liu et al., 2020). FUS combined with MBs can be used to deliver erythropoietin (EPO) to the injured area, increases the vascular permeability, promotes the recovery of neurons in the ischemic area, reduces the infarct volume, and exerts a significant neuroprotective effect (Wu et al., 2014). Ultrasound-targeted microbubble destruction (UTMD) can facilitate the delivery of NF genes into brain to protect against the development of neurodegenerative diseases (Lin et al., 2020). UTMD can be used to deliver BDNF to the brain, to promote functional recovery and white matter repair without increasing BBB damage (Rodriguez-Frutos et al., 2016). In addition, FUS can facilitate the delivery of isopropanol in the brains of epileptic rats to exert therapeutic effects against epilepsy (Airan et al., 2017). Magnetic resonance imaging-guided FUS (MRgFUS), enhances endogenous antibody delivery in AD rats to rapidly reduce the number of amyloid beta (Aβ) plaques and increase endogenous immunoglobulin levels and microglial activation (Jordao et al., 2013). MRgFUS is also a safer method used to deliver drugs across the BBB *in vivo* (Nance et al., 2014). A clinical study demonstrated that MRgFUS can be used for non-invasive, spatially targeted delivery of the monoclonal antibody trastuzumab in the brain *via* the BBB in patients with Her2-positive brain metastases (Meng et al., 2021). When cerebral ischemia occurs, the BBB will be opened, and macromolecular drugs can enter the ischemic area. However, the time and the therapeutic effect are limited due to the inability to achieve a sufficiently high dose of the drug in the infarcted area (Menzies et al., 1993). FUS combined with microbubbles can open the BBB and improve the permeability of blood vessels, thereby prolongs the time of drug treatment and improves the treatment effect. This technology has good prospects in the treatment of ischemic stroke.

## Limitations

Basic and clinical studies have demonstrated the efficacy of TFUS. It has been found to enhance drug delivery and neuroprotection. However, due to differences between animals and humans, making the effectiveness of this technology needs to be further verified. Moreover, given the diversity of stroke types, TUS may not necessarily have a beneficial effect in every patient.

The application of TFUS is limited by acoustics-related factors. Because the skull has a high attenuation coefficient, it can absorb and reflect a large amount of ultrasonic energy (Quadri et al., 2018). The absorption of ultrasonic energy can interfere with the transmission of ultrasound, preventing it from acting effectively on the target area and reducing the effectiveness of treatment. In addition, the TFUS focus is relatively small and fixed; however, it has already been demonstrated that through phased array technology, TFUS can be used to treat multiple areas in the brain (Khanna et al., 2017).

Ultrasonic energy is a double-edged sword. Application of ultrasound at an inappropriate power and at the wrong time can cause damage to brain tissue. An almost infinite range of parameters and brain regions need to be studied. Future studies are needed to determine the optimal pulse parameters and dose-response effect on region-specific neural activity (Fomenko et al., 2018). In addition, the electrophysiological and functional responses of cortical and subcortical encephalic regions to ultrasound have yet to be explored.

## Conclusion and expectation

TUS has the potential to become a clinical treatment for stroke. It can promote functional recovery from nerve recovery promotion, inflammatory response reduction, and will not cause serious tissue damage. It can also improve the curative efficacy by thrombolysis and drugs delivery. Because of the advantages of safety, non-invasiveness, deep penetration and tiny focus, it is gradually used in clinic. However, the clinical evidence regarding the efficacy of TUS is still very limited. In addition, findings from animal studies, such as those related to effective drugs and treatment parameters, may not be applicable to human beings. Especially in terms of thrombolysis, the currently approved thrombolytic drug is tPA, and the improper use of parameters will increase the risk of injury. More experiments are needed to provide strong theoretical support for the clinical application of ultrasound stimulation.

## Author contributions

LL and LY: conceptualization. LL, LY, and HH: methodology. JG and LY: writing – original draft preparation. LY, JG and WL: writing – review and editing. All authors contributed to the article and approved the submitted version.

## Conflict of interest

The authors declare that the research was conducted in the absence of any commercial or financial relationships that could be construed as a potential conflict of interest.

## Publisher’s note

All claims expressed in this article are solely those of the authors and do not necessarily represent those of their affiliated organizations, or those of the publisher, the editors and the reviewers. Any product that may be evaluated in this article, or claim that may be made by its manufacturer, is not guaranteed or endorsed by the publisher.

## Abbreviations

TUS: transcranial ultrasound stimulation
TMS: transcranial magnetic stimulation
tDCS: transcranial direct current stimulation
tACS: transcranial alternating current stimulation
tPA: tissue plasminogen activator
TFUS: transcranial focused ultrasound
PD: Parkinson’s disease
AD: Alzheimer’s disease
LIFU: low intensity focused ultrasound
LIPUS: low intensity pulsed ultrasound
BDNF: brain derived neurotrophic factor
VEGF: vascular endothelial growth factor
MCAO: middle cerebral artery occlusion
BBB: blood-brain barrier
PTUS: pulsed transcranial ultrasound stimulation.
